# Vascularized Liver‐on‐a‐Microsphere Reveals Alanine‐Glucose Metabolism‐Driven Regulation of Liver Function and Injury

**DOI:** 10.1002/advs.76211

**Published:** 2026-07-06

**Authors:** Jingyang Li, Zengnan Wu, Yingrui Zhang, Shulang Chen, Yongning Lin, Shiyu Chen, Tong Xu, Xianli Meng, Yi Zhang, Jin‐Ming Lin

**Affiliations:** ^1^ State Key Laboratory of Southwestern Chinese Medicine Resources School of Pharmacy Chengdu University of Traditional Chinese Medicine Chengdu China; ^2^ Beijing Key Laboratory of Microanalytical Methods and Instrumentation Key Laboratory of Bioorganic Phosphorus Chemistry & Chemical Biology (Ministry of Education) Department of Chemistry Tsinghua University Beijing China; ^3^ School of Ethnic Medicine Chengdu University of Traditional Chinese Medicine Chengdu China

**Keywords:** addressable hydrogel microsphere, hepatic lobule, metabolic profiling, microfluidics, vascularized liver model

## Abstract

The hepatic lobule, the basic unit of the liver, spatially organizes diverse cell types to coordinate metabolism, detoxification, and immune regulation. Yet efficiently reconstructing its hierarchical and vascular microarchitecture in vitro remains a central challenge. Here, we report a vascularized liver‐on‐a‐microsphere (VLOM) system that recreates lobule‐like structure and function within individual microscale hydrogel particles. Using a microfluidic‐aerosol fabrication strategy, compartmentalized microspheres with rough‐textured surface and built‐in spatial addressing markers were produced to spatially localize multiple liver cell types and support endothelial barrier formation. The VLOMs exhibit uniform morphology, structural stability, and high viability, accompanied by enhanced hepatic performance, including elevated albumin and urea synthesis and upregulated CYP2B6 and CYP3A4 expression. Metabolomic profiling reveals activation of the alanine‐glucose metabolic cycle, which strengthens biosynthetic and detoxification capacities. Supplementation with alanine or glucose further restores hepatic function and mitigates rifampicin‐induced hepatotoxicity, underscoring the protective role of this cycle in maintaining metabolic resilience. This scalable, vascularized microsphere system unites microengineering precision and physiological fidelity, offering a high‐throughput route to model liver metabolism and evaluate drug responses.

## Graphical abstract

A vascularized liver‐on‐a‐microsphere system is developed to recreate lobule‐like cellular organization and endothelial barrier function within compartmentalized hydrogel particles. The platform enhances hepatic performance and reveals alanine‐glucose cycle activation linked to improved detoxification capacity, offering a high‐throughput microscale model for probing liver microenvironment and drug responses.

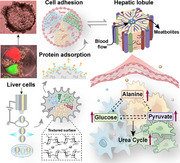

## 1 Introduction

The liver, as the largest metabolic organ in the human body, orchestrates nutrient conversion [1], detoxification [2], and immune homeostasis [3] through its fundamental structural unit—the hepatic lobule [4]. Within the lobule, diverse cell types, including hepatocytes, Kupffer cells, hepatic stellate cells, and endothelial cells form a highly ordered microarchitecture that governs metabolic flux and intercellular communication [5, 6, 7]. The integrity of this spatial hierarchy is crucial for coordinating metabolism and maintaining systemic balance [8]. However, the lack of in vitro models that recapitulate the lobule's multicellular organization and vascular interfaces continues to hinder both mechanistic studies and preclinical drug evaluation. While animal models partially reproduce hepatic physiology, species‐specific differences restrict their translational relevance [9]; moreover, ethical concerns have driven the search for alternative technologies to reduce or replace animal use in biomedical research [10]. Thus, there is a pressing need for scalable, physiologically relevant hepatic microtissues that integrate structural fidelity, metabolic functionality, and high‐throughput manufacturability.

To accurately recapitulate the complex microenvironment of the liver, significant efforts have been invested in developing diverse in vitro models [11]. Conventional two‐dimensional (2D) cell culture models are operationally simple, but they lack the three‐dimensional (3D) architecture and heterotypic signaling required for physiological responses [12]. Spheroid and organoid systems improve cell‐cell communication [13, 14]. However, they are limited by low reproducibility, long maturation time, and diffusion limitations that impede metabolic crosstalk. Recent advances in microfabrication, such as microfluidics, photolithography, and 3D printing, have enabled controlled assembly of multicellular constructs with tunable geometries [15, 16, 17]. Yet, most current designs rely on random cell encapsulation, offering limited spatial control and lacking the vascular barriers essential for directional mass transfer and functional zonation [18, 19, 20]. Consequently, reconstituting hepatic lobules with spatially organized, vascularized microdomains remains an unsolved engineering challenge.

**FIGURE 1:**
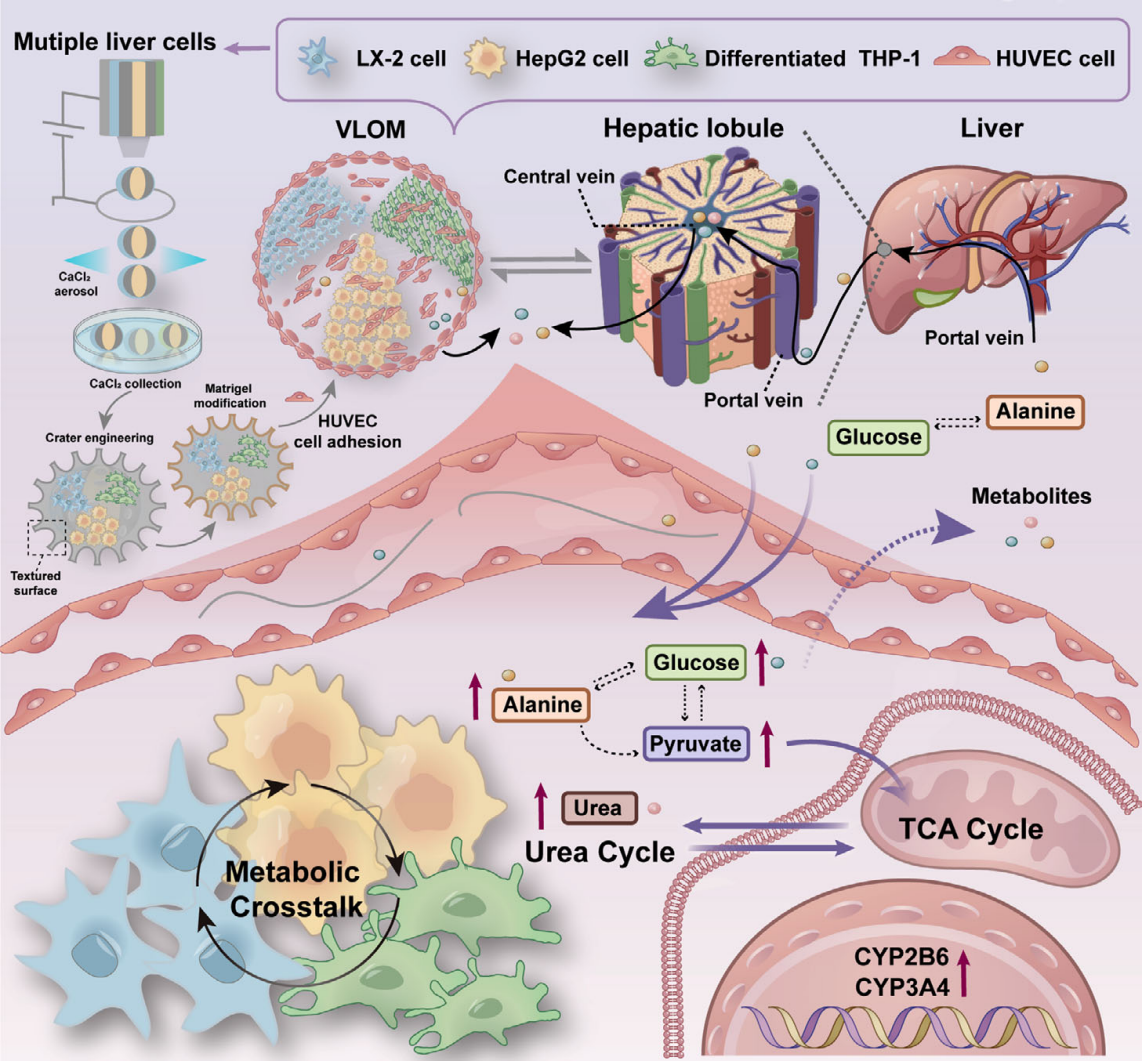
Schematic representation of a microsphere‐based hepatic lobule model for simulating cellular metabolic crosstalk within the liver microenvironment.

Here, we introduce a vascularized liver‐on‐a‐microsphere (VLOM) system that reproduces hepatic lobule‐like architecture and function within individual hydrogel particles (Figure 1). Inspired by the radial organization of native lobules, a custom microfluidic‐aerosol strategy was proposed to generate six‐compartment alginate microspheres with tunable surface roughness. The compartmentalized interiors enable the spatial encapsulation of hepatocytes (HepG2), hepatic stellate cells (LX‐2), and Kupffer‐like THP‐1 cells, thereby reconstructing parenchymal‐nonparenchymal interactions within a confined microscale space. Meanwhile, the engineered rough surface facilitates Matrigel adsorption and promotes the self‐assembly of an endothelial monolayer, forming a semi‐permeable barrier that supports directional diffusion and signal exchange akin to in vivo hepatic sinusoids.

The VLOMs exhibited uniform morphology, structural stability, and high cell viability, together with enhanced liver functions, including increased albumin and urea synthesis and upregulated CYP2B6 and CYP3A4 expression. Metabolomic analyses reveal a distinct metabolic phenotype characterized by the activation of the alanine‐glucose metabolic cycle, a key pathway coupling carbon and nitrogen metabolism. This cycle supports bidirectional alanine‐glucose conversion via pyruvate linking gluconeogenesis, glycolysis, and the urea cycle. In the multicellular environment of the microspheres, such metabolic coupling is amplified through paracrine communication, strengthening biosynthesis and detoxification capacities. Furthermore, when challenged with rifampicin‐induced hepatotoxic stress, the VLOMs maintain metabolic performance and mitigate injury markers (ALT and AST). Supplementation with alanine or glucose further restores albumin and urea production, confirming the protective role of the alanine‐glucose cycle in sustaining liver resilience. Collectively, this study establishes a scalable VLOM system that integrates structural precision, vascular functionality, and metabolic complexity. By coupling multicompartmental design with endothelial barrier formation, the platform bridges the gap between simplified cultures and physiologically relevant liver models, offering a versatile tool for drug screening and metabolic studies.

## 2 Results and Discussion

### 2.1 Fabrication of Textured Multicompartmental Microspheres

The microfluidic device for fabricating multicompartmental microspheres is composed of an upstream microfluidic chip, a downstream conductive needle, and aerosol generators (Figure S1). A device equipped with a six‐channel chip was employed to fabricate six‐compartment microspheres with rough surfaces. Multiple alginate solutions containing red or green fluorescent nanoparticles were injected into the chip as the dispersion phase. Owing to the laminar flow effect, a stable six‐compartment stream was formed and guided into the conductive needle. Under a high‐voltage electric field, droplets detached when the combined force of electric stress and gravity exceeds the surface tension. As droplets descended, they were exposed to a CaCl_2_ aerosol atmosphere created by aerosol generators. Meanwhile, aerosol collisions induced localized, non‐uniform crosslinking between Ca^2+^ and alginate, forming irregularly solidified spots on the microsphere surface. Subsequently, the textured droplets fell into a collection tank containing Ca^2+^, where full crosslinking stabilized the microspheres [21].

Fluorescence microscopic imaging revealed that the prepared microspheres exhibited highly consistent internal structures: red and green signals were uniformly distributed and confined to their predesignated compartments with negligible crosstalk (Figure S2a,b). Size analysis indicated narrow dispersity in diameter, with a coefficient of variation (CV) below 4%, confirming the successful preparation of monodisperse microspheres (Figure S2c). Furthermore, precise control over microsphere size can be achieved by fine‐tuning the fabrication parameters (Figure S2d). To visualize surface features, microspheres were prepared in nanoparticle‐free alginate, revealing a crater‐like roughness (Figure S2e).

### 2.2 Fabrication and Evaluation of the VLOMs

**FIGURE 2:**
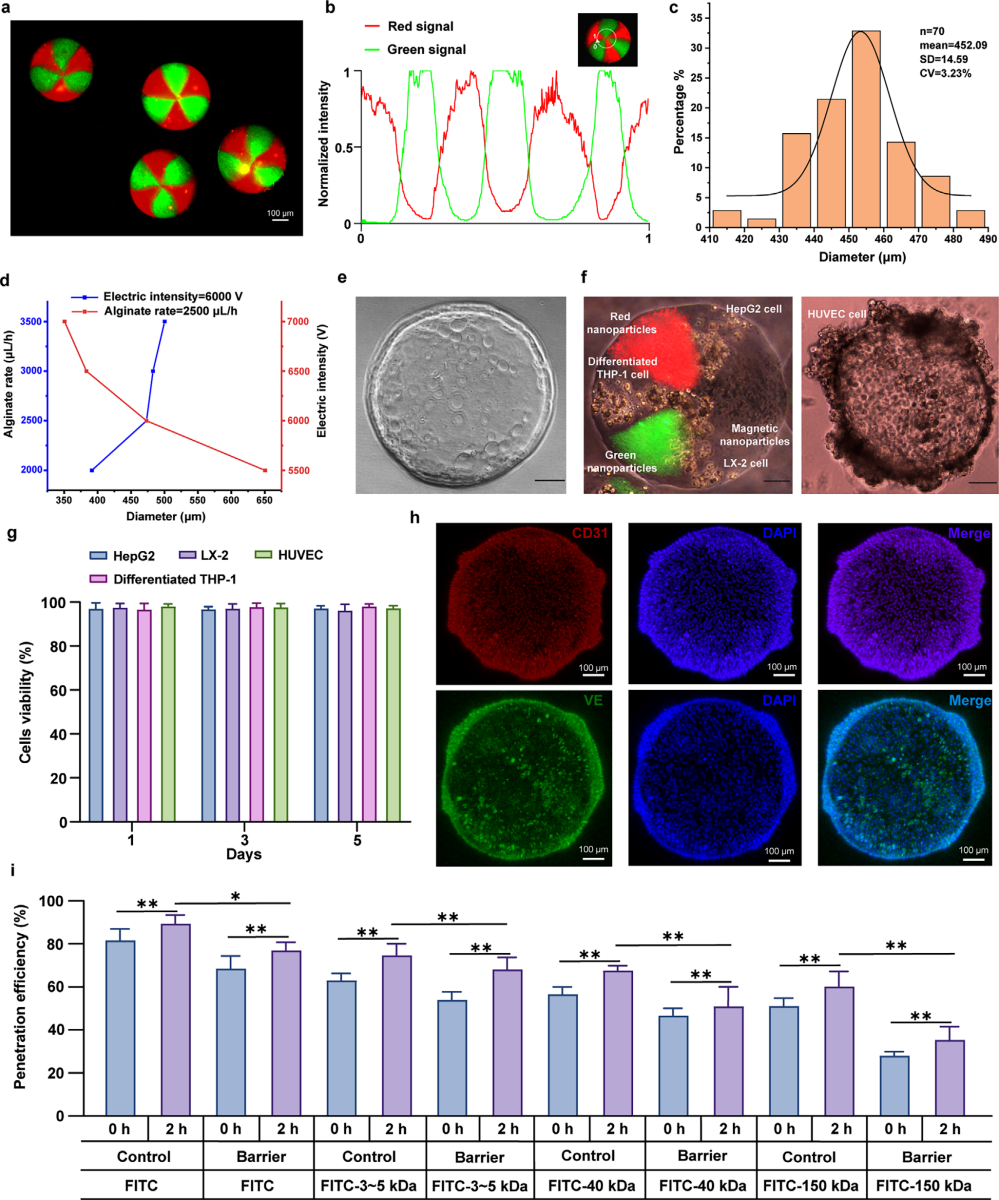
The preparation and functional characterization of VLOMs. (a) Workflow for the generation of six‐compartmental microspheres with surface cell adhesion. (b) Identification of cell types within microspheres with rough surfaces and endothelial cell adhesion. (c) Cell viability of HepG2, LX‐2, differentiated THP‐1, and HUVEC cells. (d) Confocal images of VE‐cadherin/CD31 and nucleus from the HUVEC cell layer barrier after 3days cultivation. (e) Evaluation of endothelial barrier permeability by observing the diffusion of FITC, 3–5, 40, and 150 kDa FITC‐conjugated dextran into microspheres with or without an HUVEC barrier. Data are presented as the mean ± SD; *n* = 4. Statistical significance was analyzed using unpaired two ‐tailed *t*‐test. ^*^ *p* < 0.05, ^**^ *P* < 0.01. Scale bars, 100 µm.

Following the aforementioned protocol, microfluidics was used to define internal compartments, and aerosol treatment‐generated roughened surfaces to trigger subsequent Matrigel deposition. Multiple liver‐associated cell types (HepG2 cells, LX‐2 cells, differentiated THP‐1 cells, and HUVEC cells) were integrated to construct VLOMs (Figure 2a). Differentiated THP‐1 cells can effectively recapitulate the functional characteristics of hepatic Kupffer cells (Figure S3a) [22]. To facilitate observation, one compartment was loaded with magnetic nanoparticles as a positioning marker, with the opposite compartment loaded with HepG2 cells. Adjacent compartments were seeded with LX‐2 cells and differentiated THP‐1 cells, which were respectively labeled with red and green fluorescent nanoparticles to achieve precise spatial localization (Figure S3b). This configuration established the basis for fabricating cell‐laden microspheres with rough surfaces (Figure S4a). Because rough‐textured surfaces can enhance protein adsorption [23], the cell‐laden microspheres were immersed in Matrigel for surface modification [24]. Matrigel facilitates cell adhesion via receptor‐protein interactions, enabling subsequent cell attachment [25]. Notably, rough surfaces are more effective than smooth surfaces at promoting both protein adsorption and cell adhesion, and the rate of endothelial layer formation correlates positively with the degree of surface roughness. Coating with Matrigel further amplifies this effect by enabling non‑specific protein adsorption on the rough microspheres, thereby facilitating endothelial layer formation [21]. After incubation with a HUVEC suspension, cells adhered firmly and formed a cell layer on the microsphere surfaces within three days (Figure 2b, Figure S4b,c). By day 5, the endothelial cells proliferated and established a dense endothelial barrier layer, with Hoechst live‐cell staining confirming a favorable cellular state (Figure S4d,e).

The identification of internal cell types within VLOMs was achieved by referencing magnetic nanoparticles and red/green fluorescent nanoparticle compartments (Figure S5a). Cell viability under conditions with and without an endothelial barrier was evaluated using Live/Dead cell staining. The results demonstrated that throughout the 5‐day culture period, all cell types maintained a high survival rate (>98%) in both conditions (Figure 2c; Figures S5b and S6). During prolonged culture (14 and 20 days), cell viability remained stable at above 80%. Thus, the VLOM system provides a long window of sustained cell viability (Figure S7).

VE‐cadherin is an endothelial‐specific cadherin essential for vascular homeostasis [26]; it mediates the formation of form stable cell‐cell junctions and maintains barrier integrity [27, 28]. CD31 is a widely used endothelial marker that regulates adhesion molecule and vascular homeostasis [29, 30, 31] and also orchestrates endothelial rearrangement and barrier adaptation [32]. Based on these molecular properties, we performed immunofluorescence staining for VE‐cadherin and CD31 to validate the formation of an endothelial layer on the microsphere surface. As shown in Figure 2d, CD31 staining confirmed the presence of an active endothelial barrier. Simultaneously, VE‐cadherin fluorescence was observed across the entire microsphere surface, validating the adhesion function and integrity of the endothelial barrier.

To assess HUVEC barrier function, microspheres with or without the endothelial layer were exposed to FITC and FITC‑dextran with molecular weights of 3–5, 40, and 150 kDa. The FITC‑dextran permeability assay [33] is widely used to evaluate endothelial molecular sieving [34, 35]. Free FITC (0.4 kDa) represents small metabolites and drugs; 3–5 kDa represents small signaling molecules [36] 40 kDa approximates medium proteins; 150 kDa represents large proteins and antibodies [37, 38]. Fluorescence intensities inside and outside the microspheres were measured at 0 and 2 h to assess the permeability of the endothelial barrier.

FITC and 3–5 kDa dextran showed time‐dependent penetration in both microspheres with and without HUVEC coverage, indicating that the endothelial layer allowed the transport of small molecules (Figure 2e; Figure S8). In contrast, 40 kDa dextran diffused into control microspheres within 2 h but was strongly restricted by the HUVEC layer, demonstrating the size‐selective barrier function of the endothelial coating. For 150 kDa dextran, penetration remained minimal in both groups, suggesting that large‐molecule diffusion was limited by the hydrogel matrix and was further restricted by the endothelial layer. These results indicate that the HUVEC‐covered microspheres support selective molecular permeability, allowing small‐molecule transport while suppressing macromolecular penetration. In Transwell systems, 40 and 70 kDa FITC‑dextran are standard barrier integrity indicators [39, 40]; HUVEC monolayers partially restrict FITC‐Dextran transport [41]. In microfluidic vascular models, 4 kDa dextran transport resembles Transwell, while 70 kDa shows higher permeability under dynamic condition [42]. Consistent with these models, our HUVEC barrier effectively hindered 40–150 kDa dextran, confirming its functional integrity and size‑selective semipermeability.

### 2.3 Functional Assessment of VLOMs

Hepatic parenchymal cells (e.g., HepG2) are primarily responsible for protein synthesis and metabolism [43, 44], whereas non‐parenchymal liver cells (e.g., LX‐2 cell) play a critical role in regulating microenvironmental homeostasis. These populations engage in dynamic crosstalk to collaboratively establish and modulate the liver microenvironment. HepG2 cells have been widely used as hepatic parenchymal cells in engineered liver models, including organ‑on‑a‑chip platforms [45], 3D liver spheroids [46], and in vitro hepatic lobule models [47], owing to their stable growth, reproducibility, and measurable hepatic functional readouts. Therefore, HepG2 cells were selected for proof‐of‐concept validation of the VLOM platform, including multicompartmental microsphere construction, endothelial barrier formation, dynamic culture, and metabolic analysis.

**FIGURE 3:**
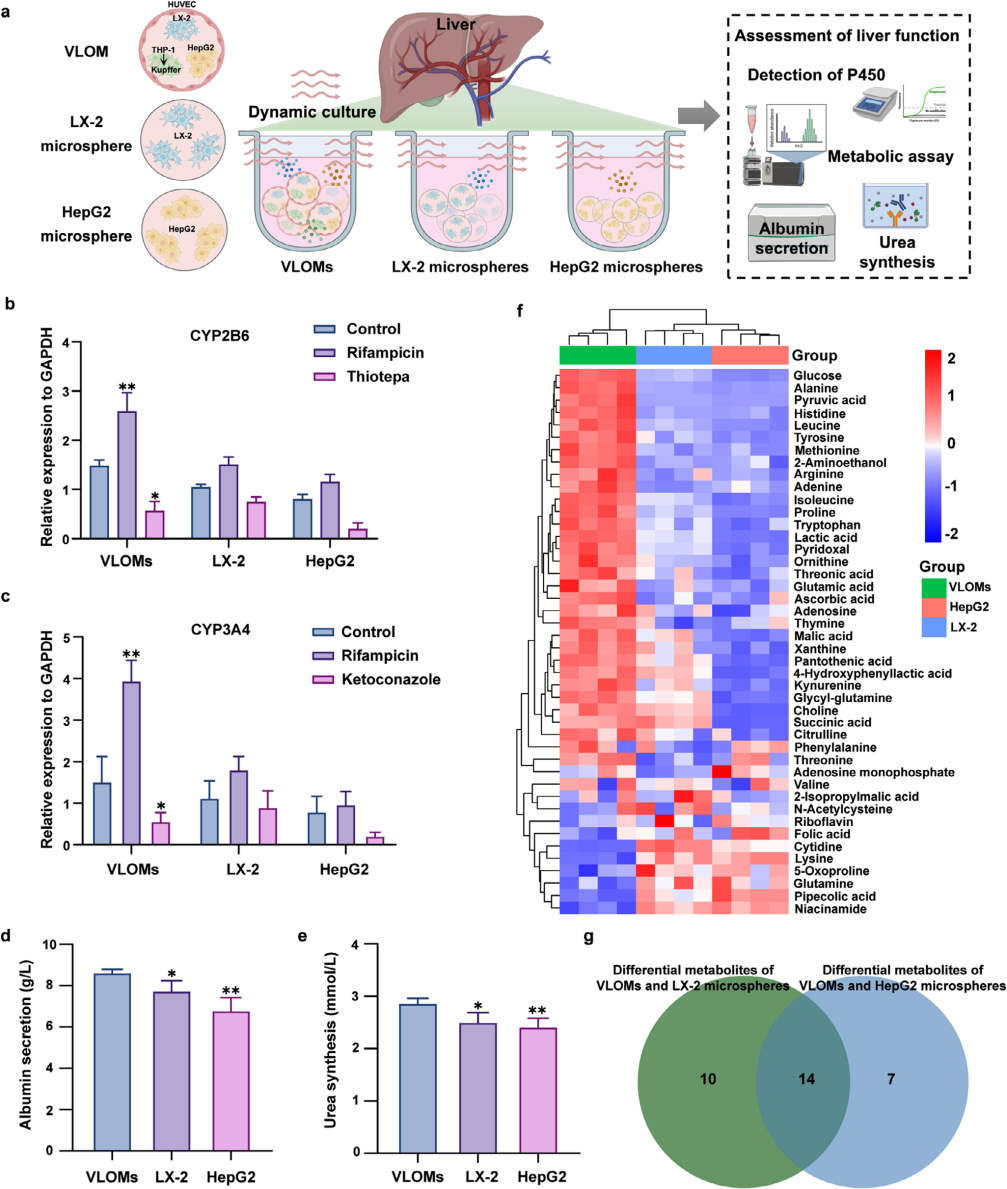
Comparative analysis of liver function between VLOMs and monocultured liver microspheres. (a) Workflow for the evaluation of liver function. qRT‐PCR was used to assess mRNA expression levels of CYP2B6 (b) and CYP3A4 (c) in VLOMs relative to monocultured liver microspheres, with GAPDH serving as the internal reference gene. Levels of albumin secretion (d) and urea synthesis (e) were also measured and compared among microspheres. (f) A hierarchical clustering heatmap was generated to visualize differentially abundant metabolites selected using t‐tests/ANOVA, with red indicating upregulation and blue indicating downregulation. (g) The number of differential metabolites in VLOMs and monocultured liver microspheres. Data are presented as the mean ± SD; *n* = 4. Statistical significance was analyzed using one‐way ANOVA followed by Tukey's multiple comparisons test. ^*^ *P*<0.05, ^**^ *P*<0.01.

This study compares the impact of co‐culture versus monoculture systems on the formation and functional sustainability of the liver microenvironment. The VLOMs, as well as the microspheres containing only LX‐2 cells or HepG2 cells, were cultured under dynamic conditions (Figure S9), and their liver‐specific functions were subsequently evaluated (Figure 3a). CYP3A4 and CYP2B6, as key members of the cytochrome P450 enzyme family, are predominantly expressed in the liver and play essential roles in hepatic metabolic processes [48]. Specifically, CYP3A4 is the most abundant drug‐metabolizing enzyme, whereas CYP2B6 is specifically involved in the biotransformation of various xenobiotics [49]. To evaluate the responsiveness of different microsphere types to enzyme modulators, low‐dose rifampicin (10 Μm) was used to induce the expression of CYP3A4 and CYP2B6 [50], while thiotepa (4 Μm) [51, 52] and ketoconazole (10 Μm) [53] served as inhibitors of CYP2B6 and CYP3A4, respectively. All dosages were within the cytocompatible range (Figures S10a,b and S11a). Upon treatment, VLOMs exhibited significant upregulation of CYP2B6 and CYP3A4 mRNA expression following agonist exposure, and markedly downregulation in response to inhibitors (Figure 3b,c). In contrast, LX‐2 and HepG2 cell microspheres showed negligible changes under the same conditions. These results demonstrate that VLOMs exhibit enhanced metabolic responsiveness to P450 modulators compared to monocultured liver microspheres, confirming their superior mimicry of hepatic metabolism.

Furthermore, albumin and urea serve as classical indicators for evaluating liver function, reflecting the liver's biosynthetic capacity [54] and detoxification metabolism [55], respectively. Albumin, synthesized by hepatocytes, is vital for maintaining colloid osmotic pressure, transporting substances, and serving as a nutritional reservoir [56, 57]. Urea, produced via the hepatic urea cycle, converts ammonia into non‐toxic compounds and serves as an essential indicator of detoxification efficiency [58]. The VLOMs exhibited significantly enhanced albumin synthesis (Figure 3d) and urea production (Figure 3e) compared with monocultured liver microspheres, demonstrating enhanced biosynthetic and metabolic performance. These findings indicated that the integrated VLOM architecture, combining a spatially organized multicellular interior with an external endothelial interface, provides stronger liver‐associated functional performance than simplified monoculture microspheres. The enhanced albumin secretion, urea production, and CYP responsiveness reflect system‐level outcomes of the complete vascularized multicellular design.

### 2.4 Metabolic Differences Between VLOMs and Monocultured Liver Microspheres

**FIGURE 4:**
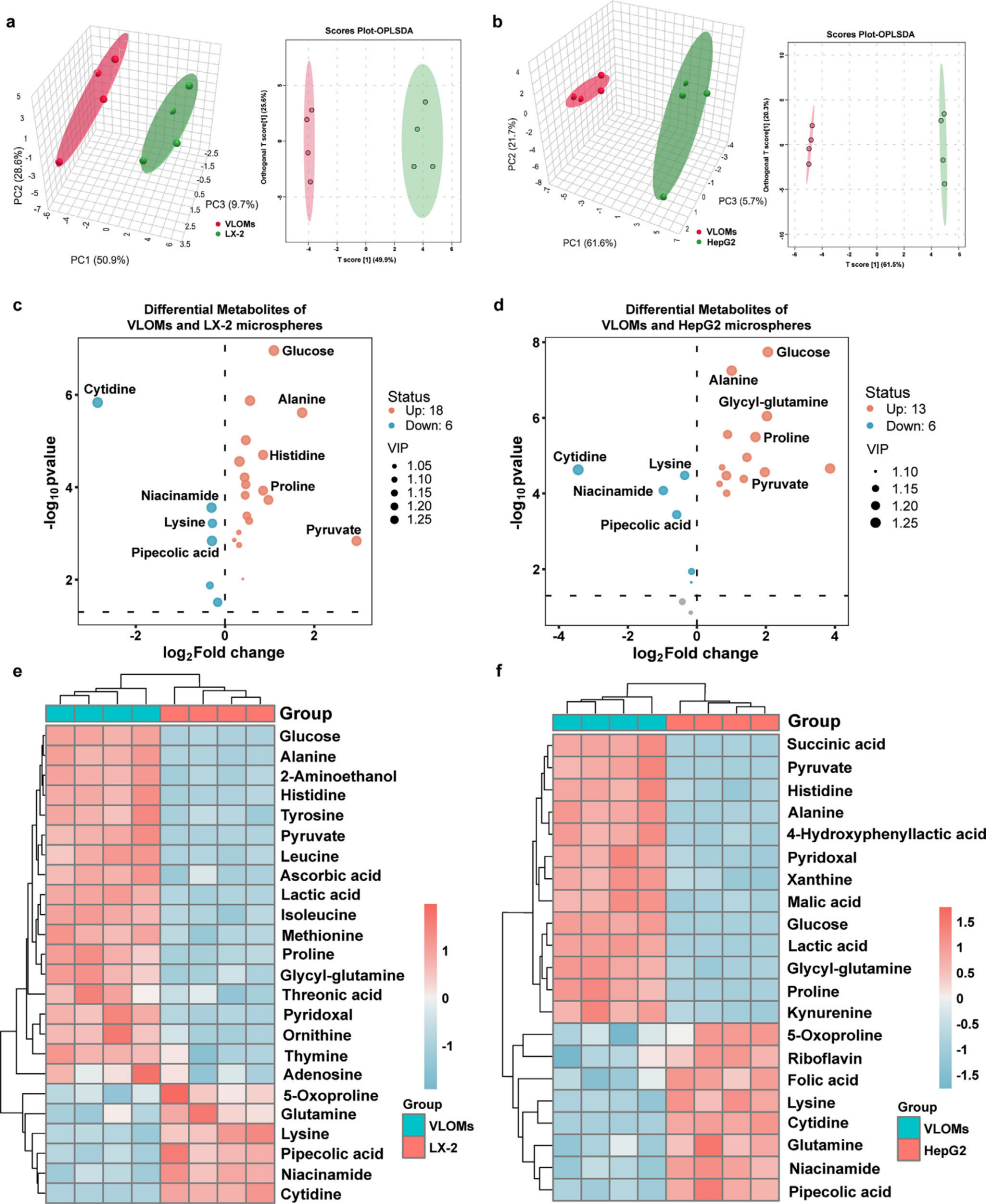
Metabolic differences between VLOMs and monocultured liver microspheres. Principal component analysis (PCA) and orthogonal partial least squares discriminant analysis (OPLS‐DA) score plots were generated to compare metabolic profiles of VLOMs with LX‐2 cell microspheres (a) and HepG2 cell microspheres (b). Volcano plot illustrating the distribution of differential metabolites between VLOMs and LX‐2 cell microspheres (c), as well as HepG2 cell microspheres (d). Cluster heatmap of differential metabolite profiles between VLOMs and LX‐2 cell microspheres (e), as well as HepG2 cell microspheres (f).

To further elucidate the metabolic differences between monocultured and VLOMs, comprehensive metabolic profiling was conducted. Notably, VLOMs showed higher metabolite levels than monocultured microspheres, with 2.67‐fold and 2.14‑fold increases over LX‐2 and HepG2 microspheres, respectively (Figure 3f). Specifically, 24 differential metabolites were identified between VLOMs and LX‐2 microspheres, and 21 between VLOMs and HepG2 microspheres, with 14 shared in both comparisons (Figure 3g). Principal component analysis (PCA) based on mass spectrometry (MS) data revealed distinct clustering patterns between the two groups, indicating substantial metabolic divergence. Orthogonal partial least squares discriminant analysis (OPLS‐DA) further confirmed significant metabolic alterations between VLOMs and monocultured liver microspheres (Figure 4a,b). Moreover, the volcano plot revealed that most upregulated differential metabolites in VLOMs were located on the right side, whereas downregulated differential metabolites were on the left side (Figure 4c,d).

In the cluster analysis comparing VLOMs with LX‑2 or HepG2 monoculture microspheres (Figure 4e,f), the differential metabolites exhibited a coordinated pattern of metabolic reprogramming. Among the upregulated metabolites, the simultaneous elevation of glucose, pyruvate, lactic acid, and other intermediates of glycolysis and gluconeogenesis suggests activation of both metabolic pathways; whereas the upregulation of tricarboxylic acid (TCA) cycle intermediates such as succinate and malic acid reflects enhanced mitochondrial oxidative metabolism [59, 60]. The marked upregulation of alanine, histidine, proline, and glycyl‑glutamine indicates that VLOMs promote the alanine‑glucose cycle, thereby providing carbon skeletons for gluconeogenesis and nitrogenous substrates for the urea cycle. Ornithine, a core intermediate of the urea cycle, was elevated, directly reflecting increased urea cycle flux [61]. Pyridoxal, a coenzyme for aminotransferases, was also elevated, supporting enhanced transamination activity and ensuring an adequate supply of ammonia for urea synthesis [62]. The increase in ascorbic acid may reflect enhanced antioxidant defense capacity, while the upregulation of aromatic metabolites such as 4‑hydroxyphenylacetic acid, xanthine, and kynurenine suggests reprogramming of phenylalanine, tyrosine, and tryptophan metabolism. Metabolites uniquely upregulated in the comparison with LX‑2 microspheres (i.e., 2‑aminoethanol, threonic acid, thymine, and adenosine) are associated with membrane remodeling and nucleotide signaling. In contrast, metabolites uniquely upregulated in the comparison with HepG2 microspheres (i.e., succinic acid, malic acid, kynurenine, and xanthine) further highlight enhanced TCA cycle flux and amino acid metabolism.

Among the downregulated metabolites, 5‑oxoproline, lysine, cytidine, glutamine, pipecolic acid, and niacinamide were commonly decreased in both comparisons. The reduction in 5‑oxoproline suggests accelerated utilization of the glutathione cycle. The decreased levels of lysine and cytidine reflect increased precursor consumption for protein synthesis and nucleotide metabolism. The decline in glutamine may indicate its rapid conversion to glutamate for entry into the TCA cycle or for ammonia detoxification pathways. Pipecolic acid, an intermediate of lysine metabolism, decreased in parallel with lysine consumption. The reduction in nicotinamide (a NAD^+^ precursor) suggests extensive NAD^+^ consumption for redox reactions and DNA repair under conditions of heightened metabolic demand. Riboflavin and folic acid, uniquely downregulated in the comparison with HepG2 microspheres, further demonstrate rapid utilization of metabolic cofactors to support energy metabolism and biosynthesis. Detailed information on the differential metabolites is provided in Tables S1 and S2. Collectively, these findings demonstrate that VLOMs exhibit a distinct and more active metabolic phenotype compared with monocultured liver microspheres. The coexistence of multiple cell types within VLOMs enhances intercellular communication and promotes the expression of distinct metabolites, thereby improving the recapitulation of native liver microenvironment.

### 2.5 The VLOMs Improve Liver Function by Enhancing the Alanine‐Glucose Metabolic Cycle

**FIGURE 5:**
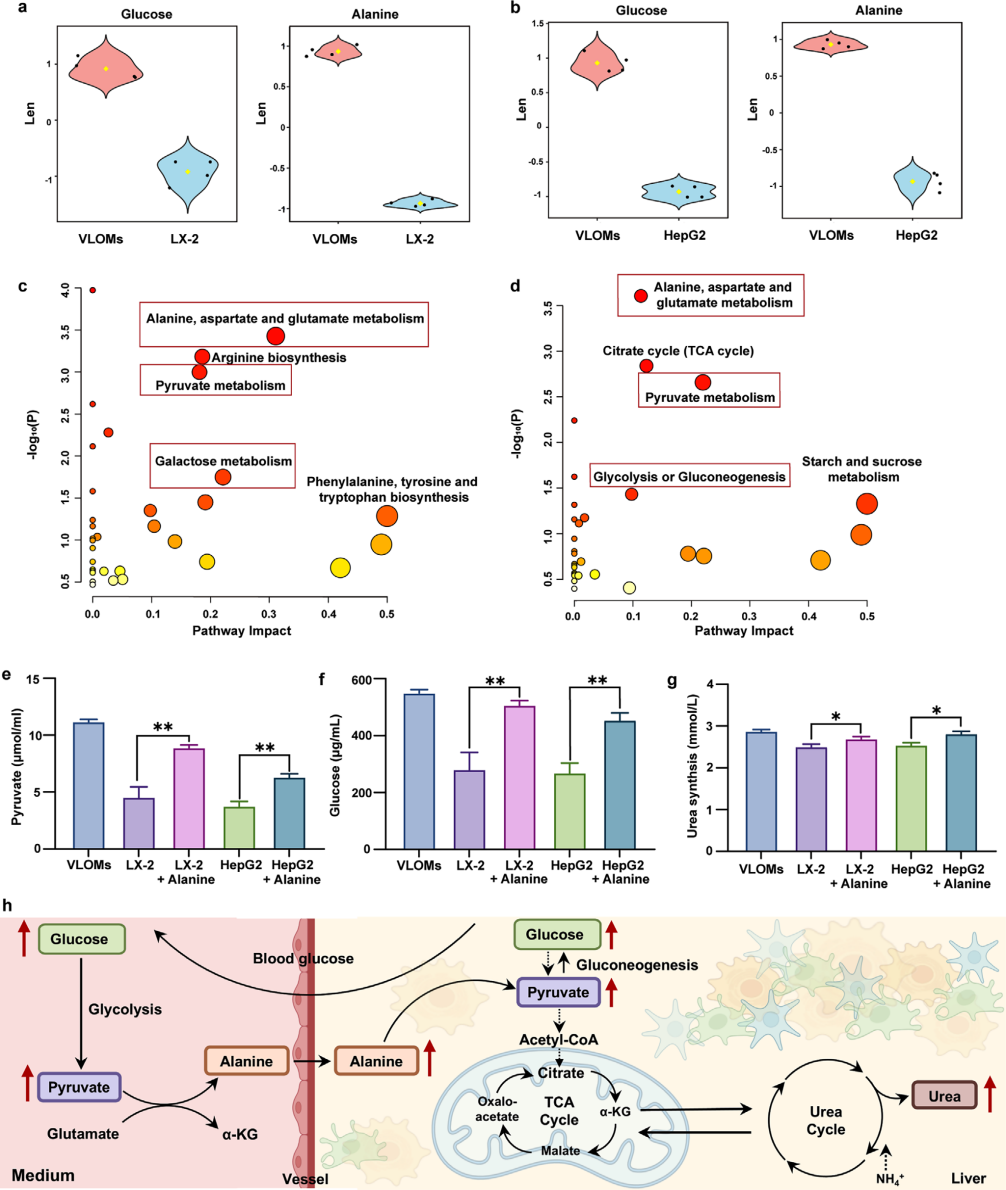
The VLOMs improve liver function by enhancing the alanine‐glucose metabolic cycle. Alanine and glucose levels exhibited significant differences between VLOMs and both LX‐2 cell microspheres (a) and HepG2 cell microspheres (b). Pathway analysis overview depicting the altered metabolic pathways in VLOMs compared to LX‐2 cell microspheres (c) and HepG2 cell microspheres (d). Quantification of (e) pyruvate, (f) glucose, and (g) urea levels in VLOMs and monocultured liver microspheres after supplementation with alanine. (h) Schematic illustration of the alanine‐glucose metabolic cycle mechanism in VLOMs. Data are presented as the mean ± SD; *n* = 4. An unpaired two ‐tailed t‐test was used in panels (e–g). ^*^ *P*<0.05, ^**^ *P*<0.01.

Among all differential metabolites, glucose and alanine exhibited the most pronounced upregulation in VLOMs compared to monocultured liver microspheres (Figure 5a,b). To further identify the underlying metabolic pathways, KEGG enrichment analysis was performed. Compared to LX‐2 cell microspheres, VLOMs significantly impacted five pathways: alanine, aspartate, and glutamate metabolism; arginine biosynthesis; pyruvate metabolism; galactose metabolism; and phenylalanine, tyrosine, and tryptophan biosynthesis (Figure 5c; Table S3). Compared to HepG2 cell microspheres, they affected: alanine, aspartate, and glutamate metabolism; TCA cycle; pyruvate metabolism; glycolysis or gluconeogenesis; and starch and sucrose metabolism (Figure 5d; Table S4). At the level of glucose metabolism, the synchronized upregulation of glycolytic/gluconeogenic and TCA cycle intermediates, including glucose, pyruvate, lactic acid, succinate, and malic acid, along with the pathway enrichment results described above, collectively indicates a globally enhanced glucose metabolic flux in VLOMs. At the level of amino acid metabolism, the increased levels of alanine, histidine, proline, glycyl‐glutamine, and ornithine are consistent with the enrichment of alanine, aspartate and glutamate metabolism and arginine biosynthesis pathways, suggesting that the alanine‑glucose cycle is amplified and that urea cycle flux is increased. Pyruvate, as a key nodal metabolite, is upregulated, and its associated pathway enrichment further supports the coupling between glucose and amino acid metabolism. Moreover, the enrichment of galactose metabolism, phenylalanine, tyrosine and tryptophan biosynthesis, as well as starch and sucrose metabolism indicate expansion of the glucose metabolic network, activation of protein synthesis, and regulation of glycogen metabolism, respectively. The functional grouping of differential metabolites in VLOMs is summarized in Table S5. These findings indicate that VLOMs possess a distinct metabolic profile, particularly characterized by promoting alanine‐glucose cycle and associated energy metabolism pathways.

To further directly verify the activity of the alanine glucose cycle in the VLOM coculture system, we supplemented the monoculture medium with exogenous alanine (8 mM, Figure S10b) and compared the downstream metabolites pyruvate (Figure 5e), glucose (Figure 5f), and urea (Figure 5g). In the absence of alanine supplementation, the basal levels of pyruvate, glucose, and urea in VLOMs were approximately 2.5‐fold, 2.0‐fold, and 1.15‐fold higher, respectively, than those in LX 2 microspheres, and similarly higher than those in HepG2 microspheres. Following alanine supplementation, pyruvate, glucose, and urea levels in LX 2 microspheres increased by approximately 2.0‐fold, 1.9‐fold, and 1.08‐fold, respectively, while those in HepG2 microspheres increased by about 1.4‐fold, 2.1‐fold, and 1.1‐fold, respectively, all showing significant increases compared with un‐supplemented conditions. These results indicate that VLOMs inherently possess a higher flux through the alanine glucose cycle, and that exogenous alanine can effectively activate this cycle and its downstream urea synthesis pathway in monoculture microspheres. In conclusion, the coculture system enhances both glucose production and urea synthesis by potentiating the alanine glucose cycle, thereby improving overall biosynthetic and metabolic functions.

From a mechanism perspective, alanine undergoes deamination in the liver to form pyruvate [63, 64], which subsequently enters the gluconeogenesis pathway for glucose production [65, 66]. Alanine is among the most efficient gluconeogenic amino acids in the liver [67]. Alanine aminotransferase (ALT), including the ALT1 and ALT2 isoforms, catalyzes the reversible conversion between alanine and pyruvate, a process that is a critical step in alanine‐driven gluconeogenesis [63]. In parallel, the liver maintains blood glucose homeostasis through glycogen synthesis and degradation, as well as ongoing gluconeogenesis [68, 69]. These findings revealed that alanine and glucose form a dynamic bidirectional regulatory network in liver metabolism. In the VLOM system, metabolomic data further confirmed the promoting effect of coculture on this cycle: alanine, pyruvate, and glucose were all significantly upregulated, and positive correlations were observed among them. Within this cycle, pyruvate is converted to glucose via gluconeogenesis, and glucose is subsequently released into the culture medium through transport mechanisms. Meanwhile, the elevated glucose can be regenerated to pyruvate through glycolysis, which, in conjunction with glutamate, further drives alanine synthesis, thereby establishing a self‑reinforcing positive metabolic feedback loop. Additionally, intracellular pyruvate can enter the TCA cycle via acetyl‐CoA to produce α‐ketoglutarate (α‐KG), thereby stimulating the urea cycle and enhancing urea metabolism. Alanine also contributes to the urea cycle by supplying NH_4_^+^, which promotes urea production and alleviates ammonia toxicity, thus strengthening the liver's metabolic detoxification capacity (Figure 5h). Recent studies have shown that hepatocyte arginase 2 (ARG2) mediates the hierarchical regulation between the urea cycle and the TCA cycle, and that ARG2 deficiency leads to defects in both urea and TCA cycle fluxes, as well as impaired mitochondrial oxidative metabolism [70]. Under conditions of increased metabolic demand, glutamine oxidation helps maintain TCA cycle flux and supports cell survival [64]. The alanine‐glucose cycle is tightly coupled with the hepatic urea cycle, and one of its key functions is to detoxify ammonium ions generated from protein catabolism via the urea cycle. The VLOMs effectively promote the glucose‑alanine cycle by enhancing intercellular metabolic communication. This cycle operates in synergistic coupling with the TCA cycle and the urea cycle, collectively improving hepatic biosynthetic and metabolic detoxification capacities. These findings further highlight the physiological relevance of multicellular microspheres in sustaining liver function in vitro.

### 2.6 The VLOMs Alleviate Liver Injury by Promoting Alanine‐Glucose Metabolic Cycle

**FIGURE 6:**
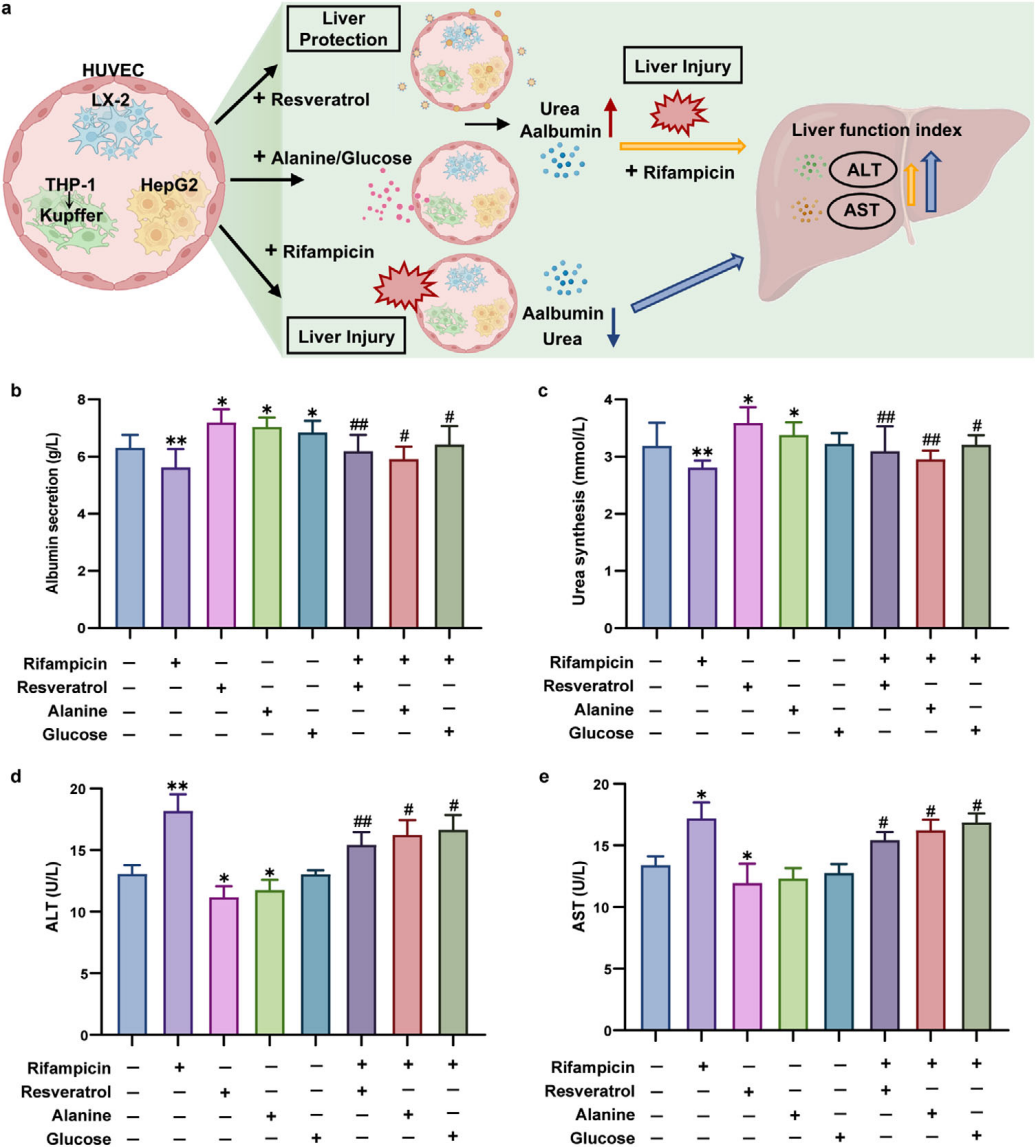
The VLOMs alleviate liver injury by promoting alanine‐glucose metabolic cycle. (a) Workflow for the assessment of liver protection and liver injury. Determine the level of albumin secretion (b), urea synthesis (c), ALT (d), and AST (e), following various treatments with rifampicin, resveratrol, and alanine or glucose supplementation in VLOMs. Data are presented as the mean ± SD; *n* = 4. Statistical significance was analyzed using one‐way ANOVA followed by Tukey's multiple comparisons test. ^*^ *P*<0.05, ^**^ *P*<0.01 compared to the control; ^#^ *P* < 0.05; ^##^ *P* < 0.01 compared to the rifampicin (+).

A liver injury model was established using high‐dose rifampicin (200 µM, Figure S10a) [71], which is sufficient to induce a clear hepatotoxic phenotype in the co‐culture system while leaving an observation window for the rescue effects of alanine and glucose [72]. Resveratrol (100 µM), a compound with known hepatoprotective properties [73, 74], served as the positive control (Figure S12a). In this system, administration of resveratrol, alanine, and glucose prior to injury induction was used to investigate their potential liver‐protective mechanisms (Figure 6a). The results demonstrated that a high‐dose rifampicin significantly decreased albumin secretion and urea production in VLOMs. Simultaneously, a high‐dose rifampicin significantly increased the expression levels of liver injury markers ALT and aspartate aminotransferase (AST) [75, 76], confirming the successful induction of hepatocellular injury. Conversely, resveratrol treatment restored albumin and urea production, suppressed ALT and AST expression, and effectively alleviated rifampicin‐induced hepatotoxicity in the VLOMs. Further studies revealed that the supplementation of alanine (8 mM, Figure S11b) and glucose (10 mM, Figure S12b) at cytocompatible concentrations significantly improved the functionality of injured VLOMs. Specifically, this improvement is evidenced by increased albumin secretion and enhanced urea production, along with a reduction in the expression levels of ALT and AST (Figure 6b–e). These results indicate that alanine‐glucose metabolism plays a critical role in enhancing the functionality of VLOMs and alleviating liver injury. Furthermore, alanine demonstrates greater metabolic efficiency than glucose, which may arise from its unique role in maintaining energy homeostasis and nitrogen transport [77, 78].

Overall, alanine‐glucose metabolism may facilitate the restoration of liver cell physiological functions and strengthen their resilience to liver drug‐induced damage. This finding not only advances our understanding of liver metabolic mechanisms but also offers novel insights into developing therapeutic strategies for liver diseases based on metabolic regulation. Future studies should aim to investigate the precise mechanisms underlying alanine and glucose metabolism, to provide a stronger theoretical foundation and technical support for clinical applications.

## 3 Conclusions

In this study, we developed VLOMs that reconstruct hepatic lobule‐like microenvironments on single hydrogel particles, uniting spatially programmed co‐cultures of multiple liver‐specific cell types with a sinusoid‐like endothelial barrier. The integrated magnetic and fluorescent fiducials enabled spatial addressing of compartments, ensuring deterministic cell localization and analysis within each microsphere. The VLOMs showed uniform morphology, stable architecture, and high cell viability, along with enhanced liver functions, characterized by increased albumin and urea synthesis, as well as upregulated CYP2B6 and CYP3A4 expression. Furthermore, we found that the VLOMs enhance biosynthetic and detoxification functions via activation of the alanine‐glucose metabolic cycle, which effectively mitigates drug‐induced (e.g., rifampicin) liver injury and supports superior hepatic performance. Supplementation with alanine or glucose further restores albumin and urea production, confirming the protective role of the alanine‐glucose cycle in maintaining liver resilience. These findings demonstrate that intercellular coordination within the VLOMs microenvironment can restore functional robustness in response to drug‐induced insults. Overall, the VLOM system integrates structural precision, vascular functionality, and metabolic relevance in a scalable format, offering a versatile tool for studying liver metabolism, drug toxicity, and intercellular communication.

Future optimization of the VLOM platform will focus on incorporating more physiologically relevant parenchymal cell sources, such as primary human hepatocytes or differentiated HepaRG cells [79], to strengthen hepatic metabolic capacity, improve drug‐metabolism predictability, and expand its translational potential. The vascular architecture can also be further refined by introducing endothelial cells into defined inner compartments or arranging endothelial and parenchymal cells in multilayered configurations, thereby creating more biomimetic endothelial–parenchymal interfaces. Such spatially controlled designs may improve nutrient transport, metabolic waste exchange, and tissue‐level perfusion, supporting the development of VLOMs toward more predictive liver microphysiological systems.

## 4 Experimental Section

### 4.1 Materials

Sodium alginate and 12‐O‐tetradecanoylphorbol‐13‐acetate (PMA) were purchased from Sigma‐Aldrich (Mississippi, USA). Matrigel was obtained from Corning (New York, USA). Normal saline (0.9%), calcium chloride solution (1M), Dulbecco's Modified Eagle Medium (DMEM), Roswell Park Memorial Institute (RPMI)‐1640, penicillin‐streptomycin, trypsin, and phosphate buffered solution (PBS) were supplied by Solarbio (Beijing, China). Fetal bovine serum (FBS) was purchased from ExCell Bio (Shanghai, China). DAPI staining solution, cell counting kit‐8 (CCK‐8), and Hoechst 33342 staining solution for liver cells were obtained from Beyotime (Shanghai, China). The Live/Dead cell viability kit, CD31 monoclonal antibody, Alexa Fluor 488‐ and Alexa Fluor 555‐conjugated secondary antibodies, and fluorescent microspheres were purchased from Thermo Fisher Scientific (Massachusetts, USA). VE‐cadherin monoclonal antibody was obtained from Invitrogen (California, USA). FITC‐labeled dextran (3000–5000, 40 000, and 150 000 Da) was obtained from MedChemExpress (New Jersey, USA). Rifampicin (R006875), ketoconazole (R015536), thiotepa (R032877), glucose (CAS: 50‐99‐7), alanine (CAS:56‐41‐7) were obtained from Rhawn (Shanghai, China). Acetonitrile was obtained from Vokai Biotechnology Co., Ltd. (Beijing, China). Ultrapure water was prepared using a Milli‐Q water purification system from Millipore.

### 4.2 Installation of a Microfluidic Device

The device consists of a microfluidic chip, conductive needle, and aerosol injectors. The microfluidic chip was fabricated via soft lithography technology and features six fluidic channels with axisymmetric geometry converging at a central fluid intersection. A conductive needle was connected at the fluid intersection points. Under the combined effects of electric stress and gravity, droplets were separated from the microfluidic network and fell vertically along the flow direction. Subsequently, a rough surface was formed by spraying aerosol components. During this process, the droplets were impacted by aerosols exhibiting side cross‐linking effects, causing surface deformation. Simultaneously, the cross‐linkable components in the aerosol underwent in situ cross‐linking reactions with the hydrogel, resulting in the formation of rough textures on the droplet surfaces. Finally, the semi‐finished droplets were collected by a collector containing cross‐linking agents to further stabilize their internal structure.

### 4.3 Generation of the Six‐Compartmental Microspheres with a Rough Surface

The six‐channel integrated microfluidic device was employed to fabricate six‐compartmental microspheres. A 1.5% alginate solution was mixed with normal saline at a volume ratio of 9:1 to form an independent dispersion phase. During the experiment, the volumetric flow rate for each of the six dispersion phases was set to 2500 µL/h, and the electric field strength was adjusted to 6000 V. Meanwhile, the CaCl_2_ aerosol ejectors immediately generated a CaCl_2_ mist. Subsequently, microspheres with roughened surfaces were collected in the collecting bath under the influence of the CaCl_2_ mist.

### 4.4 Cell Culture and Differentiation

The human hepatic stellate cells (LX‐2), human hepatocarcinoma cells (HepG2) and human umbilical vein endothelial cells (HUVEC) were cultured in DMEM supplemented with 10% FBS, 1% penicillin, and streptomycin under 5% CO_2_ at 37°C. Human acute monocytic leukemia cells (THP‐1) were grown in RPMI‐1640 supplemented with 10% FBS and 1% penicillin and streptomycin. Subsequently, THP‐1 cells were harvested and resuspended in RPMI‐1640 medium supplemented with 100 ng/mL PMA for 24 h to induce differentiation, which served as a substitute for Kupffer cells.

### 4.5 Fabrication of the VLOM and Monoculture Microspheres

All microspheres (VLOM and monoculture) were fabricated using the same microfluidic chip with matched total cell numbers. For VLOM microsphere, HepG2, LX‑2, differentiated THP‑1 cells, and HUVEC cells were each prepared at a density of 1 × 10^7^ cells/mL. For monoculture microsphere (HepG2 or LX‑2), cells were prepared at a density of 2.5 × 10^7^ cells/mL. Each microsphere contained three cell‑encapsulating compartments: VLOM contained three distinct cell types (one per compartment), while monocultures contained the same cell type in all compartments. During fabrication, the 1.5% alginate solution containing cells in a 9:1 volume ratio was injected into the microfluidic system. Meanwhile, an equal volume of magnetic particles, blue nanoparticles, and green nanoparticles were mixed with alginate in the same proportion for cell localization. CaCl_2_ aerosol ejectors were subsequently employed to spray along the dripping path of the droplets for the preparation of a rough surface. For surface modification of the microspheres, they were transferred into a solution containing 0.5 mg/mL Matrigel for cell adhesion experiments. HUVEC cells were seeded onto the microsphere surfaces and co‐incubated for 2 h. A fresh culture medium supplemented with 5 mM CaCl_2_ was then added, and the cell‐laden microspheres were further incubated at 37 °C and 5% CO_2_ for subsequent tests. Monocultures were treated identically except that they were not coated with HUVEC cells.

### 4.6 Viability Assay

On days 1, 3, and 5, cell viability was assessed using the Live/Dead Cell Double Staining Kit. The microspheres were incubated with the Calcein‐AM/PI working staining solution for 20 min, and fluorescence was subsequently observed via confocal microscopy. Cell viability was quantified using ImageJ software and calculated as follows: viability (%) = number of live cells/total number of cells.

### 4.7 Vascular Endothelial Barrier Functionality Assay

To visualize and quantify the endothelial barrier function, the expression of VE‐cadherin, a protein associated with tight junctions between adjacent cells, was verified by immunostaining. For immunofluorescence staining, the VLOMs were harvested after 3 days of culture and washed three times with normal saline containing 0.1 M CaCl_2_. They were then fixed in 100 µL of 4% paraformaldehyde for 15 min at room temperature. The supernatant was subsequently removed, and the microspheres were washed three times before being permeabilized with 100 µL of 0.5% Triton X‐100 for 10 min. After washing three times again, the microspheres were incubated with 100 µL of blocking solution for 1 h at room temperature. Primary antibodies against VE‐Cadherin and CD31 were diluted at 1:100 and incubated with the microspheres overnight at 4°C. Following this, the microspheres were washed and incubated with secondary antibodies, goat anti‐rabbit IgG H&L (Alexa Fluor 488) and goat anti‐mouse IgG H&L (Alexa Fluor 555), which were diluted at 1:500 and incubated for 1 h at 37°C. Finally, the nuclei were stained with DAPI for 10 min, and fluorescence images were acquired using confocal microscopy. In addition, solutions containing FITC‐labeled dextran (0, 3–5 kDa, 40, and 150 kDa) were prepared at a concentration of 25 µg/mL in DMEM supplemented with 5 mM CaCl_2_. Subsequently, the microsphere reservoirs with and without the HUVEC cell layer were incubated in the solutions for 2 h. The diffusional permeability was evaluated by monitoring the diffusion of FITC‐labeled probes into the hydrogel microspheres. To correct for initial differences in fluorescence intensity, normalization was performed using the penetration efficiency, defined as the ratio of internal to external fluorescence intensity (FI_inside_ / FI_outside_), where FI_inside_ and FI_outside_ represent the fluorescence intensities inside and outside the microspheres, respectively. All images were processed using ZEN software (Zeiss, Germany).

### 4.8 RNA Extraction and Quantitative Real‐Time PCR (qRT‐PCR)

Total RNA was extracted from VLOMs, LX‐2 microspheres, and HepG2 microspheres using an ABclonal RNA isolation Kit (Wuhan, China). The concentration of the RNA was measured using a NanoDrop 2000 spectrophotometer (Thermo Scientific, USA). cDNA was synthesized using a reverse transcription kit for qPCR (ABclonal, Wuhan, China). Fast qPCR Mix (ABclonal, Wuhan, China) was used for qRT‐PCR analysis. The expression levels of target genes were normalized to those of GAPDH. The primers used in this study were as follows: CYP3A4, forward: 5’‐ TGAGGCGGGAAGCAGAGA ‐3’; reverse: 5’‐ CATGCTGTAGGCCCCAAAGA ‐3’. CYP2B6, forward: 5’‐ ATGGGGCACTGAAAAAGACTGA ‐3’; reverse: 5’‐ AGAGGCGGGGACACTGAATGAC ‐3’. GAPDH, forward: 5’‐CCTGGTATGACAACGAATTTG‐3’; reverse: 5’‐CAGTGAGGGTCTCTCTCTTCC‐3’.

### 4.9 Cytotoxicity Assay

The LX‐2, HepG2, HUVEC and differentiated THP‐1 cells in the logarithmic growth phase (1×10^5^ cells/mL) were incubated in a 96‐well plate for 24 h and then stimulated with different concentrations of rifampicin, thiotepa, ketoconazole, and resveratrol (0, 20, 50, 100, 200, 400, 800, 1000 µm), as well as alanine and glucose (0, 1, 2, 4, 8, 10, 20, 40 mm) for 24 h. Then 10 µL of CCK‐8 reagent was added to each well, and the cells were incubated for 1.5 h. Subsequently, the optical density (OD) at 450 nm was measured using a microplate reader (Thermo Fisher Scientific, USA).

### 4.10 Measurement of Extracellular Cytokines

Cytokines associated with liver metabolism were quantitatively measured. The concentrations of albumin (A028‐2‐1), urea (C013‐1‐1), alanine (C009‐2‐1), pyruvate (A081‐1‐1), alanine aminotransferase (C009‐1‐1), and aspartate aminotransferase (C010‐2‐1) in the culture medium were determined using corresponding assay kits (Nanjing Jiancheng Bioengineering Institute, China). Meanwhile, the extracellular glucose concentration in the culture medium was quantified using the Glucose Assay Kit (Sigma‐Aldrich).

### 4.11 Chromatographic and Electrospray Ionization Mass Spectrometry Conditions

In this experiment, a C18 column (100 mm × 2.1 mm, 3 µm; Shimadzu, Japan) was mounted on a liquid chromatography triple quadrupole tandem mass spectrometry system (LC‐QqQ‐MS/MS, Shimadzu, Japan). The temperature of the column was set to 40°C. The mobile phase was prepared with 0.1% (v/v) aqueous formic acid (solvent A) and 0.1% (v/v) formic acid in acetonitrile (solvent B), delivered at a flow rate of 0.3 mL/min. Gradient elution was performed according to the following schedule: 0% B (0‐1.4 min), 0%–25% B (1.4–3.5 min), 25%–35% B (3.5–7.5 min), 35%–95% B (7.5–10.3 min), 95% B (10.3–13.7 min), and 95%–0% B (13.7–13.8 min). The supernatant of the microspheres was collected and precipitated with acetonitrile at a volume ratio of 1:2. The mixture was then vortexed and centrifuged at 10 000 rpm for 10 min to remove salts and proteins. Protein concentrations in each sample were quantified to normalize metabolite abundance. The samples were stored in an autosampler at 4°C with an injection volume of 2 µL. Mass spectrometric parameters were set as follows: interface temperature: 300°C, heating block temperature: 250°C, desolation line temperature: 150°C, spray voltage: 3.00 kV, nebulizer gas (N_2_) flow rate: 3 L/min, and heating gas (N_2_) and drying gas (N_2_) flow rate: 10 L/min. Each metabolite was detected in positive or negative ion mode using multiple reaction monitoring.

### 4.12 Statistical Analysis

Raw data were processed according to the requirements of each assay. Fluorescence‐based permeability data were normalized using the penetration efficiency, defined as the ratio of internal to external fluorescence intensity. All data were expressed as the mean ± standard deviation (SD) of at least three independent experiments. The difference between two groups was analyzed using unpaired two‐tailed t‐test. Multiple group comparison was performed using one‐way analysis of variance (ANOVA) followed by Tukey's multiple comparisons test. Statistical difference is pointed as ^*^ *P* < 0.05, ^**^ *P* < 0.01, ^***^ *P* < 0.001; ^#^ *P* < 0.05, ^##^ *P* < 0.01, ^###^ *P* < 0.001. All statistical analyses were performed using Origin 2021 and GraphPad Prism 9.

## Author Contributions

J.L.: conceptualization, methodology, investigation, validation, formal analysis, writing – original draft. Z.W.: conceptualization, methodology, writing – review & editing, supervision, project administration. Y.Z.: data curation, formal analysis. S.C.: methodology. Y.L.: methodology. S.C.: data curation. T.X.: formal analysis. X.M.: formal analysis. Y.Z.: supervision, project administration. J.L.: conceptualization, methodology, supervision, project administration.

## Funding

This work was supported by the National Key Research and Development Program of China (No. 2024YFB4607800), the National Natural Science Foundation of China (No. U23A20520, 22404094, 22034005) and the Natural Science Foundation of Sichuan Province (No. 2024NSFSC0701).

## Conflicts of Interest

The authors declare no conflicts of interest.

## Supporting information




**Supporting File**: advs76211‐sup‐0001‐SuppMat.docx.

## Data Availability

The data that supports the findings of this study are available in the supplementary material of this article.
