# Supplementary material for: Vascularized Liver‐on‐a‐Microsphere Reveals Alanine‐Glucose Metabolism‐Driven Regulation of Liver Function and Injury
Source: Adv Sci (Weinh). 2026 Jul 6;13(60):e76211. doi: 10.1002/advs.76211 (PMC13335659; doi:10.1002/advs.76211)
Supplement: Supplementary file 1 [file ADVS-13-e76211-s001.docx]

Supporting Information

Vascularized liver-on-a-microsphere reveals alanine-glucose metabolism-driven regulation of liver function and injury

Jingyang Li, Zengnan Wu*, Yingrui Zhang, Shulang Chen, Yongning Lin, Shiyu Chen, Tong Xu, Xianli Meng, Yi Zhang*, and Jin-Ming Lin*

J. Li, Y. Zhang, S. Chen, T. Xu, X. Meng, Y. Zhang.

State Key Laboratory of Southwestern Chinese Medicine Resources, School of Pharmacy, Chengdu University of Traditional Chinese Medicine, Chengdu, 611137, China.
E-mail: [zhangyi@cdutcm.edu.cn](mailto:zhangyi@cdutcm.edu.cn)

Z. Wu, S. Chen, Y. Lin, J. Lin

Beijing Key Laboratory of Microanalytical Methods and Instrumentation, Key Laboratory of Bioorganic Phosphorus Chemistry & Chemical Biology (Ministry of Education), Department of Chemistry, Tsinghua University, Beijing, 100084, China.

E-mail: [wzn.2021@tsinghua.org.cn](mailto:wzn.2021@tsinghua.org.cn), [jmlin@mail.tsinghua.edu.cn](mailto:jmlin@mail.tsinghua.edu.cn)

Y. Zhang

School of Ethnic Medicine, Chengdu University of Traditional Chinese Medicine, Chengdu, 611137, China

**This file contains 11 supplementary figures and 4 supplementary tables.**

**Supplementary figures:**

**Supplemental figure 1**. Photographs of the microfluidic device for six-compartmental microspheres production. (a) From left to right, the components include the cell injection pump, the six-chamber microfluidic device equipped with a conductive needle, and the voltage controller. (b) The conductive needle and magnetic field. (c) Microfluidic device for generating sodium alginate microparticles with rough surfaces.

**Supplemental figure 2**. The preparation and functional characterization of six-compartmental vascularized cell-cocultured liver microsphere. (a) A microscopic image of fluorescent microspheres. (b) Fluorescence intensity analysis of each compartment in microspheres. (c) Size distribution of the generated microspheres. (d) Adjustable sizes of microspheres by regulating electric intensity or alginate rate. (e) Bright field images of microspheres with rough surfaces. Scale bar, 100 μm.

**Supplemental figure 3**. Differentiation of THP-1 cells and microsphere-based cell encapsulation. (a) The differentiation of THP-1 cells into macrophages was induced through a 24 h treatment with 12-O-tetradecanoylphorbol-13-acetate (PMA). (b) Cell encapsulation utilizing microsphere-based technology and the spatial localization of three types of liver-related cells (HepG2, LX-2 and differentiated THP-1 cell) through green/red nanoparticles. Scale bars, 50 μm.

**Supplemental figure 4**. The adhesion of endothelial cells to six-compartmental microspheres with rough surface. (a) Six-compartmental microspheres containing encapsulated cells with rough surface. (b) Adhesion of endothelial cells (HUVEC) to the surface of six- compartmental cell microspheres. (c) The inverted dish was utilized for cell adhesion, with sterile water added to maintain humidity and prevent excessive evaporation of the cell suspension. (d) On the third, fifth, and seventh days following the initiation of HUVEC cell adhesion and growth. (e) Hoechst staining of live cells on the seventh day of HUVEC cell adhesion. Scale bars, 50 μm.

**Supplemental figure 5**. Assessment of cell localization and viability within VLOMs. (a) Cell localization within the six- compartmental microsphere following HUVEC cell adhesion. Scale bars, 50 μm. (b) Fluorescence imaging of cell viability levels following 1-, 3-, and 5-days cultivation. Scale bars, 100 μm.

**Supplemental figure 6**. Cell viability of liver microsphere without HUVEC cell adhesion. Cell viability rate of HepG2, LX-2, and differentiated THP-1 cells. Data are presented as the mean ± SD; n = 4. Scale bars, 50 μm.

**Supplemental figure 7**. Cell viability of VLOMs at days 14 and 20. Cell viability rate of HepG2, LX-2, differentiated THP-1 cells, and HUVEC cells. Data are presented as the mean ± SD; n = 4. Scale bars, 50 μm.

**Supplemental figure 8**. Evaluation of endothelial barrier permeability by observing the diffusion of FITC, 3~5, 40 and 150 kDa FITC-conjugated dextran into microspheres with or without an HUVEC barrier. Scale bars, 100 μm.

**Supplemental figure 9**. The microspheres were dynamic cultured through cyclic propulsion and suction of the culture medium at 40 μl/h, with six microspheres per well.

**Supplemental figure 10**. Cell viability of LX-2, HepG2, differentiated THP-1 and HUVEC cells following treatment with rifampicin (a) and thiotepa (b). Data are presented as the mean ± SD; n = 4. Statistical significance was analyzed using one-way ANOVA followed by Tukey’s multiple comparisons test. ^*^*P*＜0.05, ^**^*P*＜0.01 compared to the control (0 μM).

**Supplemental figure 11**. Cell viability of LX-2, HepG2, differentiated THP-1 and HUVEC cells after treatment with ketoconazole (a) and L-alanine (b). Data are presented as the mean ± SD; n = 4. Statistical significance was analyzed using one-way ANOVA followed by Tukey’s multiple comparisons test. ^*^*P*＜0.05, ^**^*P*＜0.01 compared to the control (0 μM or 0 mM).

**Supplemental figure 12**. Cell viability of LX-2, HepG2, differentiated THP-1 and HUVEC cells after treatment with resveratrol (a) and glucose (b). Data are presented as the mean ± SD; n = 4. Statistical significance was analyzed using one-way ANOVA followed by Tukey’s multiple comparisons test. ^*^*P*＜0.05, ^**^*P*＜0.01 compared to the control (0 μM or 0 mM).

**Supplementary tables:**

Table S1 Differential metabolites between VLOMs and LX-2 microspheres.

| **Metabolite name** | **log_2_FC** | **P-value** | **VIP** | **Regulation** |
| --- | --- | --- | --- | --- |
| Glucose | 1.095198325 | 1.10708E-07 | 1.2698135 | Up |
| Alanine | 1.728450543 | 2.44135E-06 | 1.2595723 | Up |
| 2-Aminoethanol | 0.466137028 | 9.59111E-06 | 1.2288100 | Up |
| Histidine | 0.852169557 | 2.00415E-05 | 1.2364770 | Up |
| Tyrosine | 0.308965022 | 0.000948821 | 1.0477319 | Up |
| Pyruvic acid | 2.94681731 | 0.00144997 | 1.2386441 | Up |
| Leucine | 0.439090741 | 6.11081E-05 | 1.2124662 | Up |
| Ascorbic acid | 0.489225348 | 0.00041678 | 1.1623885 | Up |
| Lactic acid | 0.559858919 | 1.34309E-06 | 1.2578613 | Up |
| Methionine | 0.464082432 | 8.72032E-05 | 1.1934603 | Up |
| Proline | 0.97237409 | 0.000188094 | 1.2219375 | Up |
| Glycyl-glutamine | 0.540407729 | 0.00053173 | 1.1297274 | Up |
| Threonic acid | 0.398071131 | 0.009632993 | 1.0198761 | Up |
| Pyridoxal | 0.450919094 | 0.000149161 | 1.1677281 | Up |
| Ornithine | 0.315422795 | 0.001781846 | 1.0631653 | Up |
| Adenosine | 0.852964991 | 0.000118364 | 1.2168308 | Up |
| 5-Oxoproline | -0.164909223 | 0.030746879 | 1.1929694 | Down |
| Glutamine | -0.339079794 | 0.013274019 | 1.12600594 | Down |
| Lysine | -0.283347163 | 0.00060349 | 1.19454321 | Down |
| Pipecolic acid | -0.275065708 | 0.000824275 | 1.24856535 | Down |
| Niacinamide | -0.30204352 | 0.000275893 | 1.24891190 | Down |
| Cytidine | -2.254412829 | 1.47306E-06 | 1.26744517 | Down |

Table S2 Differential metabolites between VLOMs and HepG2 microspheres.

| **Metabolite name** | **log_2_FC** | **P-value** | **VIP** | **Regulation** |
| --- | --- | --- | --- | --- |
| Succinic acid | 0.88931951 | 2.74734E-06 | 1.205061995 | Up |
| Pyruvic acid | 3.858713698 | 2.18048E-05 | 1.24745108 | Up |
| Histidine | 0.85358075 | 3.36407E-05 | 1.22688567 | Up |
| Alanine | 1.985875898 | 1.63622E-06 | 1.25417752 | Up |
| 4-Hydroxyphenyllactic acid | 1.448843644 | 1.10953E-05 | 1.21283293 | Up |
| Pyridoxal | 0.728521274 | 2.03943E-05 | 1.13632201 | Up |
| Xanthine | 0.86092778 | 9.81265E-05 | 1.15676754 | Up |
| Malic acid | 1.358108511 | 4.11544E-05 | 1.18237347 | Up |
| Glucose | 1.753272895 | 1.61895E-08 | 1.26184159 | Up |
| Lactic acid | 1.042157573 | 2.20085E-08 | 1.25301865 | Up |
| Glycyl-glutamine | 1.509123589 | 4.16286E-06 | 1.24982869 | Up |
| Proline | 1.597209439 | 2.07127E-05 | 1.23845821 | Up |
| Kynurenine | 0.655586814 | 5.57355E-05 | 1.13814393 | Up |
| 5-Oxoproline | -0.155945539 | 0.011496284 | 1.14694872 | Down |
| Riboflavin | -0.179810929 | 0.140551182 | 1.10729096 | Down |
| Folic acid | -0.423433661 | 0.072511041 | 1.14189773 | Down |
| Lysine | -0.305859769 | 7.38045E-05 | 1.21037323 | Down |
| Cytidine | -5.441092848 | 2.35704E-05 | 1.26762249 | Down |
| Glutamine | -0.163456224 | 0.022335291 | 1.09985546 | Down |
| Niacinamide | -0.341207804 | 0.000118776 | 1.20827175 | Down |
| Pipecolic acid | -0.446067025 | 8.66742E-05 | 1.21317240 | Down |

Table S3 Pathway enrichment analysis of differential metabolites identified in the VLOMs compared to LX-2 microspheres.

| **Pathway name** | **Match status** | **-log (P)** | **Holm P** | **FDR** |
| --- | --- | --- | --- | --- |
| Valine, leucine and isoleucine biosynthesis | 3/8 | 3.9733 | 0.0085064 | 0.0085064 |
| Alanine, aspartate and glutamate metabolism | 4/28 | 3.4274 | 0.029524 | 0.014949 |
| Arginine biosynthesis | 3/14 | 3.1827 | 0.05122 | 0.017511 |
| Pyruvate metabolism | 4/36 | 2.985 | 0.07262 | 0.02068 |
| Nitrogen metabolism | 2/6 | 2.6182 | 0.18309 | 0.038545 |
| Glutathione metabolism | 3/28 | 2.2802 | 0.39342 | 0.069941 |
| Glyoxylate and dicarboxylate metabolism | 3/32 | 2.1149 | 0.56804 | 0.087728 |
| Galactose metabolism | 2/16 | 1.7496 | 1.0 | 0.17798 |
| Neomycin, kanamycin and gentamicin biosynthesis | 1/2 | 1.5814 | 1.0 | 0.23303 |
| Arginine and proline metabolism | 2/23 | 1.4498 | 1.0 | 0.28399 |
| Glycolysis or Gluconeogenesis | 2/26 | 1.3513 | 1.0 | 0.32388 |
| Phenylalanine, tyrosine and tryptophan biosynthesis | 1/4 | 1.2859 | 1.0 | 0.34518 |
| Lysine degradation | 2/30 | 1.2384 | 1.0 | 0.35543 |
| Glycine, serine and threonine metabolism | 2/33 | 1.1645 | 1.0 | 0.36518 |
| Cysteine and methionine metabolism | 2/33 | 1.1645 | 1.0 | 0.36518 |
| Pyrimidine metabolism | 2/39 | 1.0377 | 1.0 | 0.43813 |
| Valine, leucine and isoleucine degradation | 2/40 | 1.0189 | 1.0 | 043813 |
| Phenylalanine metabolism | 1/8 | 0.9957 | 1.0 | 0.43813 |
| Tyrosine metabolism | 2/24 | 0.98274 | 1.0 | 0.43813 |
| Vitamin B6 metabolism | 1/9 | 0.94726 | 1.0 | 0.45165 |
| Biotin metabolism | 1/10 | 0.9042 | 1.0 | 0.47497 |
| Butanoate metabolism | 1/15 | 0.74154 | 1.0 | 0.6307 |
| Nicotinate and nicotinamide metabolism | 1/15 | 0.74154 | 1.0 | 0.6307 |
| Starch and sucrose metabolism | 1/18 | 0.67036 | 1.0 | 0.67162 |
| Ubiquinone and other terpenoid-quinone biosynthesis | 1/19 | 0.64954 | 1.0 | 0.67162 |
| Selenocompound metabolism | 1/20 | 0.62992 | 1.0 | 0.67162 |
| Citrate cycle (TCA cycle) | 1/20 | 0.62992 | 1.0 | 0.67162 |
| Purine metabolism | 2/70 | 0.62881 | 1.0 | 0.67162 |
| beta-Alanine metabolism | 1/21 | 0.61138 | 1.0 | 0.67502 |
| One carbon pool by folate | 1/26 | 0.53179 | 1.0 | 0.7818 |
| Histidine metabolism | 1/27 | 0.51802 | 1.0 | 0.7818 |
| Lipoic acid metabolism | 1/28 | 0.50484 | 1.0 | 0.7818 |
| Porphyrin metabolism | 1/31 | 0.46845 | 1.0 | 0.82437 |

Table S4 Pathway enrichment analysis of differential metabolites identified in the VLOMs compared to HepG2 microspheres.

| **Pathway name** | **Match status** | **-log (P)** | **Holm P** | **FDR** |
| --- | --- | --- | --- | --- |
| Alanine, aspartate and glutamate metabolism | 4/28 | 3.6056 | 0.019839 | 0.019839 |
| Citrate cycle (TCA cycle) | 3/20 | 2.8396 | 0.1143 | 0.057872 |
| Pyruvate metabolism | 3/23 | 2.6582 | 0.17137 | 0.058589 |
| Glyoxylate and dicarboxylate metabolism | 3/32 | 2.2405 | 0.44258 | 0.11496 |
| Neomycin, kanamycin and gentamicin biosynthesis | 1/2 | 1.6246 | 1.0 | 0.37975 |
| Glycolysis or Gluconeogenesis | 2/26 | 1.4319 | 1.0 | 0.48134 |
| Riboflavin metabolism | 1/4 | 1.3285 | 1.0 | 0.48134 |
| Starch and sucrose metabolism | 2/30 | 1.3176 | 1.0 | 0.48134 |
| Arginine and proline metabolism | 236 | 1.1751 | 1.0 | 0.55689 |
| Nitrogen metabolism | 1/6 | 1.1573 | 1.0 | 0.55689 |
| Pyrimidine metabolism | 2/39 | 1.1138 | 1.0 | 0.55968 |
| Vitamin B6 metabolism | 1/9 | 0.98855 | 1.0 | 0.68447 |
| Biotin metabolism | 1/10 | 0.94523 | 1.0 | 0.6981 |
| Arginine biosynthesis | 1/14 | 0.8088 | 1.0 | 0.82604 |
| Butanoate metabolism | 1/15 | 0.78125 | 1.0 | 0.82604 |
| Nicotinate and nicotinamide metabolism | 1/15 | 0.78125 | 1.0 | 0.82604 |
| Histidine metabolism | 1/16 | 0.75564 | 1.0 | 0.82604 |
| Lysine degradation | 1/18 | 0.7093 | 1.0 | 0.84906 |
| Purine metabolism | 2/70 | 0.69444 | 1.0 | 0.84906 |
| Selenocompound metabolism | 1/20 | 0.66834 | 1.0 | 0.84906 |
| bata-Alanine metabolism | 1/21 | 0.64954 | 1.0 | 0.84906 |
| Propanoate metabolism | 1/22 | 0.63173 | 1.0 | 0.84900 |
| One carbon pool by folate | 1/26 | 0.5687 | 1.0 | 0.84906 |
| Folate biosynthesis | 1/27 | 0.55467 | 1.0 | 0.85208 |
| Galactose metabolism | 1/27 | 0.55467 | 1.0 | 0.85208 |
| Lipoic acid metabolism | 1/28 | 0.54125 | 1.0 | 0.85208 |
| Glutathione metabolism | 1/28 | 0.54125 | 1.0 | 0.85208 |
| Glycine, serine and threonine metabolism | 1/33 | 0.48166 | 1.0 | 0.90997 |
| Cysteine and methionine metabolism | 1/33 | 0.48166 | 1.0 | 0.90997 |
| Tryptophan metabolism | 1/41 | 0.40503 | 1.0 | 1.0 |
| Tyrosine metabolism | 1/42 | 0.39788 | 1.0 | 1.0 |

Table S5 Pathway enrichment analysis of differential metabolites identified in the VLOMs compared to HepG2 microspheres.

| **Metabolic pathways** | **Metabolite** | **Expression in VLOMs** | **Biological significance** |
| --- | --- | --- | --- |
| Glucose metabolism | Glucose, pyruvate, lactic acid, | ↑ | Glycolysis/glycogenesis; TCA cycle;  Energy metabolism |
|  | Succinic acid, malic acid | ↑  (Only HepG2) | Glycolysis/glycogenesis; TCA cycle |
|  | 2-Aminohexanol; threonic acid | ↑  (Only LX-2) | Cell membrane phospholipid synthesis;  Glycosidic acid metabolism related |
| Amino acid metabolism | Alanine, histidine, proline,  glycyl-glutamine | ↑ | Alanine-glucose cycle; gluconeogenesis;  urea cycle |
|  | Ornithine, pyridoxal | ↑ | Urea cycle; ammonia detoxification |
|  | Leucine, isoleucine, methionine, tyrosine | ↑  (Only LX-2) | Amino acid metabolism; protein synthesis |
|  | 5-Oxoproline | ↓ | Glutathione cycle and γ-glutamyl cycle |
|  | Lysine, picolinic acid | ↓ | Lysine catabolic; protein synthesis |
|  | Kynurenine | ↑  (Only HepG2) | Tryptophan metabolic pathway;  immune regulation |
|  | 4-Hydroxyphenyllactic acid | ↑  (Only HepG2) | Tyrosine metabolism |
| Nucleotide metabolism | Thymine, adenosine | ↑  (Only LX-2) | Nucleotide synthesis |
|  | Xanthine | ↑  (Only HepG2) | Purine metabolism; energy metabolism |
| Co-factors and Vitamin Metabolism | Nicotinamide | ↓ | Redox reactions and DNA repair |
|  | Riboflavin | ↓  (Only HepG2) | TCA cycle |
|  | Folic acid | ↓  (Only HepG2) | One-carbon metabolism; nucleotide synthesis |
|  | Pyridoxal | ↑ | Urea production |
|  | Ascorbic acid | ↑  (Only LX-2) | Antioxidant defense; oxidative stress |
